# Structural and functional changes of bioactive proteins in donor human milk treated by vat-pasteurization, retort sterilization, ultra-high-temperature sterilization, freeze-thawing and homogenization

**DOI:** 10.3389/fnut.2022.926814

**Published:** 2022-09-15

**Authors:** Ningjian Liang, Jeewon Koh, Bum Jin Kim, Gulustan Ozturk, Daniela Barile, David C. Dallas

**Affiliations:** ^1^Nutrition Program, School of Biological and Population Health Sciences, College of Public Health and Human Sciences, Oregon State University, Corvallis, OR, United States; ^2^Department of Food Science and Technology, University of California, Davis, Davis, CA, United States

**Keywords:** heat treatment, pressure, microbiological safety, lactation, preterm infant

## Abstract

**Background:**

Donor human milk should be processed to guarantee microbiological safety prior to infant feeding, but this process can influence the structure and quantity of functional proteins.

**Objective:**

The aim of this study was to determine the effect of thawing, homogenization, vat-pasteurization (Vat-PT), retort sterilization (RTR) and ultra-high-temperature (UHT) processing on the structure of bioactive proteins in donor milk.

**Methods:**

Pooled donor milk was either not treated (Raw) or treated with an additional freeze-thaw cycle with and without homogenization, Vat-PT, RTR with and without homogenization, and UHT processing with and without homogenization. Overall protein retention was assessed *via* sodium-dodecyl sulfate (SDS-PAGE), and the immunoreactivity of 13 bioactive proteins were assessed *via* enzyme-linked immunosorbent assay (ELISA).

**Results:**

Freeze-thawing, freeze-thawing plus homogenization and Vat-PT preserved all the immunoglobulins (sIgA/IgA, IgG, IgM) in donor milk, whereas RTR and UHT degraded almost all immunoglobulins. UHT did not alter osteopontin immunoreactivity, but Vat-PT and retort decreased it by ~50 and 70%, respectively. Freeze-thawing with homogenization, Vat-PT and UHT reduced lactoferrin's immunoreactivity by 35, 65, and 84%, respectively. Lysozyme survived unaltered throughout all processing conditions. In contrast, elastase immunoreactivity was decreased by all methods except freeze-thawing. Freeze-thawing, freeze-thawing plus homogenization and Vat-PT did not alter polymeric immunoglobulin receptor (PIGR) immunoreactivity, but RTR, RTR plus homogenization and UHT increased detection. All heat processing methods increased α-lactalbumin immunoreactivity. Vat-PT preserved all the growth factors (vascular/endothelial growth factor, and transforming growth factors β1 and β2), and UHT treatments preserved the majority of these factors.

**Conclusion:**

Different bioactive proteins have different sensitivity to the treatments tested. Overall, Vat-PT preserved more of the bioactive proteins compared with UHT or RTR. Therefore, human milk processors should consider the impact of processing methods on key bioactive proteins in human milk.

## Introduction

Human milk is the optimal nutrition source for newborn infants, especially for preterm infants. Premature infants' lack of physiological development at birth leaves them at increased risk for poor growth, poor neurological development and infections. Compared with feeding infant formula, feeding preterm infants mother's milk reduces risk of necrotizing enterocolitis (1), sepsis (1) and features predictive of metabolic syndrome (2, 3). The American Academy of Pediatrics recommends that preterm infants be fed with mother's milk for the first 6 months of life (4). However, when mother's own milk is not available or not sufficient, processed donor milk is recommended to feed preterm infants (4).

Human milk contains a variety of components that have a profound role in infant survival, development and health. Among these, milk proteins contribute to many potential functions, including enhancing mineral absorption [e.g., lactoferrin (5) and α-lactalbumin (6, 7)], controlling nutrient absorption [e.g., bile salt-stimulated lipase (8) and elastase (9)], defending against bacterial and viral pathogens [e.g., lactoferrin (10, 11), lysozyme, immunoglobulins (12) and haptocorrin (13)], modulating the immune system [e.g., cytokines (14), polymeric immunoglobulin receptor (15) and osteopontin (16)] and guiding the development of the gastrointestinal system [e.g., transforming growth factor β (17) and lactoferrin (17, 18)].

Consuming donor milk provided by milk banks and by donor milk processing companies is an alternative option for preterm infants whose parents cannot produce sufficient parent's own milk. To ensure the microbiological safety, a variety of processing protocols have been developed for pasteurization of donor milk. Vat pasteurization, which is equivalent to Holder pasteurization (Vat-PT, 62.5 °C for 30 min), is the most commonly used method for human milk processing. Vat-PT destroys vegetative microorganisms in donor milk. Some donor milk is processed using retort sterilization (RTR, 121°C, 15 to 20 pounds per square inch of pressure for 5 min) and ultra-high-temperature (UHT) (130 to 140°C for 2–10 s). RTR and UHT destroy vegetative microorganisms and spores in donor milk. Donor milk is also typically exposed to freeze-thaw cycles during processing. Some processors use pressure-based homogenization after heat processing of donor milk to prevent fat separation in the finished product. Many studies have investigated the effects of different processing methods on the properties of donor milk protein. For example, previous studies investigated the effects of Holder pasteurization on proteins (19–24), immune components (25) and growth factors (24) in human milk. However, there is a lack of systematic comparison of thawing, homogenization, Holder pasteurization (equivalent to Vat-PT), RTR and UHT processing on the preservation of an array of bioactive proteins in human milk. Currently, donor milk processors lack information on optimal processing of human milk to ensure safety while maintaining bioactive protein structures. Identification of how different thermal processing methods, thawing and homogenization affect bioactive protein structure and function is critically important because this information could provide guidance to achieve precision milk protein fortification. The aim of this study was to determine the effect of freeze-thawing, homogenization, Vat-PT, RTR and UHT on the structure and function of bioactive proteins in human milk.

## Materials and methods

### Donor human milk and processing

A pool of raw, frozen human milk was collected and provided by Prolacta Bioscience (City of Industry, CA). All samples were collected within 6 months prior to testing. The first fraction of this pool was saved as its original form at −20 °C (labeled as Raw). The second fraction was thawed at 4°C for 72 h and refrozen at −20°C (labeled as Thaw Raw). The third fraction was thawed, homogenized (2,500 psi at 55°C) and refrozen at −20°C, with three independent processing replicates (labeled as Thaw Raw H 1, Thaw Raw H 2, and Thaw Raw H 3). The fourth fraction was pasteurized by Prolacta Bioscience using Vat-PT (62.5°C for 30 min) (labeled as Vat-PT). The fifth fraction was processed in the Milk Processing Lab of University of California, Davis by UHT (142°C for 6 s) in triplicate without homogenization (labeled as UHT 1, UHT 2, and UHT 3), or by UHT with homogenization (142°C for 3 s and 2,500 psi at 55°C) (labeled as H-UHT 1, H-UHT 2, and H-UHT 3). The sixth fraction was processed at North Carolina State University by RTR (121°C, 20 pounds per square inch pressure for 5 min) in triplicate without homogenization (labeled as RTR 1, RTR 2, and RTR 3), or with homogenization (labeled as H-RTR 1, H-RTR 2, and H-RTR 3). In total, 18 samples were generated for the study. The summary of the processing conditions and labeling of the processed milk samples are illustrated in Supplementary Figure S1.

### Sodium dodecyl sulfate–polyacrylamide gel electrophoresis

An aliquot of each sample was thawed at 4°C overnight. Samples were agitated using a vortex mixer for 5 s and an aliquot (100 μl) of each sample was collected to measure the protein content of whole samples (without defatting). Nine-hundred microliters of each sample were centrifuged at 4,000 × *g* for 20 min at 4°C. The infranatant (400–600 μl) between the top lipid layer and the bottom precipitate was collected and centrifuged at 4,000 × *g* for 20 min at 4°C. Three-hundred microliters of the second infranatant were collected to measure the protein content of the defatted samples. Aliquots (10 μl) from each sample collected before (whole) centrifugation and from the second infranatant (defatted) were diluted separately in 190 μl 18.2 MΩ.cm water. The concentrations of protein were determined by the Pierce™ BCA Protein Assay Kit (Thermo Fisher Scientific, Waltham, MA) according to the manufacturer's instructions. An aliquot of the second infranatant of each sample type (Raw, Vat-PT, UHT 1, H-UHT 2, RTR1, H-RTR 2, Thaw Raw and Thaw Raw H 3) was diluted in 18.2 MΩ.cm water with a dilution factor of 6–11 to load 12.5 μg of protein in each well (dilution factor based on protein concentration determination by BCA). Each diluted sample (20 μl) was mixed with 6.6 μl of 4x Laemmli loading buffer and 2.75 μl of 1 M dithiothreitol and heated at 95°C for 5 min to break disulfide bonds for complete protein unfolding. After cooling, an aliquot (20 μl) of the sample was loaded on a 12% Criterion™ TGX™ Precast Midi Protein Gel (Bio-Rad Laboratories, Hercules, CA). An aliquot (10 μl) of Precision Plus Protein™ Unstained standard (Bio-Rad Laboratories, Hercules, CA) was loaded into another lane of the gel as a molecular-weight marker. Electrophoresis was run at 200 V for 45 min in a Criterion™ cell (Bio-Rad) with 1x running buffer (Tris/Glycine/SDS). The gel was stained in Bio-Safe™ Coomassie Stain (Bio-Rad) for 1 h and distained in 18.2 MΩ.cm water for 30 min.

### Analysis of bioactive proteins

The concentration of IgA, IgG, IgM, lactoferrin, lysozyme, α-lactalbumin, tumor necrosis factor α (TNFα), polymeric immunoglobulin receptor (PIGR), osteopontin, vascular/endothelial growth factor (VEGF), transforming growth factor-β1 (TGF-β1), transforming growth factor-β2 (TGF-β2) and elastase in the donor human milk samples were determined in duplicate using the following ELISA kits: IgA Human ELISA Kit (Invitrogen, catalog # BMS2096), IgG Human ELISA Kit (catalog BMS2091, Invitrogen, Waltham, MA) and IgM Human ELISA Kit (catalog BMS2098, Invitrogen, Waltham, MA), Human LTF/Lactoferrin ELISA Kit (catalog EH309RB, Invitrogen, Waltham, MA), Human Lysozyme, LZM/LSZ ELISA Kit (catalog MBS164375, Mybiosource, San Diego, CA), Human α-Lactalbumin (aLA) ELISA Kit (catalog MBS2702818, Mybiosource, San Diego, CA), Human TNF-α Human ELISA Kit (catalog BMS223-4, Invitrogen, Waltham, MA), Human Polymeric Immunoglobulin Receptor (PIGR) ELISA Kit (catalog MBS2022400, Mybiosource, San Diego, CA), Osteopontin Human ELISA Kit (catalog BMS2066, Invitrogen, Waltham, MA), VEGF Human ELISA Kit (catalog KHG0111, Invitrogen, Waltham, MA), TGF-β1 Human ELISA Kit (catalog BMS249-4, Invitrogen, Waltham, MA), TGF-β2 Human ELISA Kit (catalog EHTGFB2, Invitrogen, Waltham, MA) and PMN (Neutrophil) Elastase Human ELISA Kit (catalog BMS269, Invitrogen, Waltham, MA). Preliminary tests were performed to determine the optimal dilution values for detection of each protein (Table 1).

**Table 1:** Detection efficiency (%) of spiked proteins in raw diluted donor human milk.

**Targeted protein**	**Dilution of the milk sample^a^**	**Spiked protein**	**Average of the detected total target protein**	**Target protein in non-spiked samples**	**Detection efficiency (%)^b^ (average ±SD)**
IgA	4,000	25 ng/ml	54.2	38.0	64.9 ± 14.5
IgG	200	250 ng/ml	269.8	87.7	73.9 ± 1.0
IgM	800	1,000 ng/ml	777.6	105.6	67.2 ± 24.0
Lactoferrin	10,000	192 ng/ml	274.7	66.0	108.7 ± 70.8
Lysozyme	500	40 ng/ml	78.8	40.6	95.7 ± 2.9
α-Lactalbumin	5,000	50 ng/ml	35.0	7.1	58.9 ± 4.3
TNF-α	0	250 pg/ml	139.9	0.0	55.9 ± 1.6
PIGR	10	400 pg/ml	71.2	0.0	17.8 ± 0.1
Osteopontin	2,500	60 ng/ml	189.8	127.7	103.6 ± 9.4
VEGF	50	750 ng/ml	1017.5	224.9	105.7 ± 6.4
TGF-β1	4.8	1,000 pg/ml	369.7	181.3	18.8 ± 0.7
TGF-β2	12	100 pg/ml	330.8	297.5	33.2 ± 33.7
Elastase	50	2.5 ng/ml	3.2	0.7	99.5 ± 3.2

a Raw donor human milk sample.

b Two replicates of spiking experiments were conducted to determine the detection efficiency.

Spiking experiments were performed to determine the detection efficiency of each protein in donor human milk. The targeted bioactive protein standard (provided in each ELISA kit) was spiked into diluted raw milk (specific dilution for each protein listed in Table 1) to reach a specific final concentration. The detection efficiency (%) was calculated as average of detected bioactive protein concentration in the diluted raw milk samples spiked with the bioactive protein–average of detected bioactive protein concentration in the diluted raw milk samples without spiking/spiked bioactive protein concentration × 100. The final concentration for each bioactive protein was adjusted using the detection efficiency (%) obtained from the spiking experiment. All the spiking experiments were performed in replicate and standard deviation were calculated based on the data obtained from the replicates. Analyses for all ELISAs were carried out following the manufacturer's protocol and standard curves were created for each analyte. All measurements were performed in replicate.

### TGF-β1-specific handling

TGF-β1 required acid activation before ELISA analysis. Specifically, 50 μl of each whole milk sample were mixed with 150 μl of assay buffer, and 20 μl of 1N HCl were added. The solutions were agitated on a vortex mixer and incubated for 1 h at room temperature. The mixtures were neutralized by addition of 20 μl 1N NaOH. The neutralized samples were added to the ELISA plates for analysis following the manufacturer's instructions.

### Statistical analysis

One-way analysis of variance (ANOVA) with *post hoc* Tukey's tests, using Graph Pad Prism software, version 8.2, were used to identify significant (*p* < 0.05) differences between samples. Data are shown as the average ± standard deviation.

## Results

### Protein concentration

The concentrations of fat-soluble proteins and whey soluble proteins were compared and shown in Supplementary Table S1. The protein concentration of each infranatant of the 18 samples were lower (*p* < 0.05) than their corresponding whole sample. In whole samples (with lipid), H-RTR had the highest protein content (14.4 ± 1.0 mg/ml), followed by Thaw Raw H (14.0 ± 0.5 mg/ml), H-UHT (13.0 ± 0.9 mg/ml), UHT (11.9 ± 1.5 mg/ml), Thaw Raw (11.3 ± 0.0 mg/ml), Raw (10.7 ± 0.1 mg/ml), RTR (9.8 ± 2.9 mg/ml) and Vat-PT (9.7 ± 0.1 mg/ml). In the infranatant (without lipid), there was no significant difference between Raw, Vat-PT, Thaw Raw, Thaw Raw H, UHT and H-UHT, but RTR and H-RTR had lower (*p* < 0.05) protein content compared with the other samples.

### SDS-PAGE

**Figure 1:**
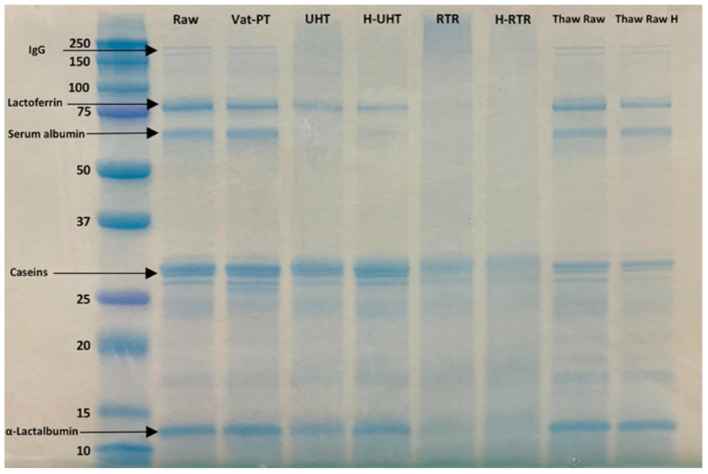
SDS-PAGE of raw milk (Raw), vat-pasteurized milk (Vat-PT), ultra-high-temperature processed milk (UHT), homogenized and ultra-high-temperature processed milk (H-UHT), retort processed milk (RTR), homogenized and retort processed milk (H-RTR), thawed raw milk (Thaw Raw), and homogenized thawed raw donor human milk (Thaw Raw H). The unit of the y-axis is kDa.

SDS-PAGE was conducted to examine differences in the overall visual protein profile of donor milk across each processing method. To investigate whether the presence of lipids would interfere with SDS-PAGE, samples (Raw, RTR 2, RTR 3, H-RTR 2, and H-RTR 3) with and without lipid were compared *via* SDS-PAGE (Supplementary Figure S2). Samples with lipid had an extra band on the bottom of the lane compared with samples without lipid. However, there was no other significant difference in banding among the samples. The identity of the extra band in lipid-containing samples was not determined. Therefore, subsequent SDS-PAGE was conducted using samples without lipid. Three batches of Thaw Raw H, UHT, and H-UHT were compared, and there were no visual differences in the density of banding among batches within the same treatment type (Supplementary Figure S3). The electrophoretic banding of all samples, including Raw, Vat-PT, UHT, H-UHT, RTR, H-RTR, Thaw Raw, and Thaw Raw H, were compared (Figure 1). In Raw, there were two thin bands between 250 and 150 kDa, which were possibly immunoglobulins such as sIgA (150 kDa) or xanthine oxidase (270 kDa). There were two distinct bands around 75 to 50 kDa, which were most likely lactoferrin (78 kDa) and serum albumin (69 kDa). Caseins were present between 37 and 25 kDa, and α-lactalbumin between 15 and 10 kDa. Vat-PT, Thaw Raw and Thaw Raw H had protein profiles similar to those of Raw. In UHT and H-UHT, two bands between 250 and 150 kDa and one band between 75 and 50 kDa that were present in Raw were absent. Moreover, RTR and H-RTR samples lacked the two bands between 250 and 150 kDa, two bands around 75 to 50 kDa and one band between 10 and 15 kDa, and had fainter bands between 37 and 25 kDa.

### Detection efficiency (%) of proteins

Spike and recovery tests were conducted to determine the extent to which the milk matrix interfered with protein binding to the capture and detect antibodies used in each immunoassay. The detection efficiency (%), that is the percentage of spiked protein recovered, for lactoferrin, osteopontin, VEGF and elastase were near 100%, indicating that there was little interference from the milk matrix with the binding of these proteins to the antibodies used in the immunoassay. In contrast, the detection efficiencies for PIGR, TGF-β1 and TGF-β2 were 17.8, 18.8, and 33.2%, respectively. This low detection efficiency demonstrated that the milk matrix blocked the majority of the proteins from binding to the antibodies used in the immunoassay. The detection efficiencies for IgA, IgG, IgM, α-lactalbumin, TNF-α were 64.9, 73.9, 67.2, and 58.9%, respectively, suggesting that the milk matrix blocked a small portion of the proteins from binding to the antibodies used in the immunoassay. These values were used to correct the protein concentrations for each sample obtained from ELISA. The corrected values approximate the “true” concentrations of targeted analytes in milk samples.

### Effect of processing methods on the concentrations of immunoglobulins (IgA, IgG and IgM) in donor human milk

**Figure 2:**
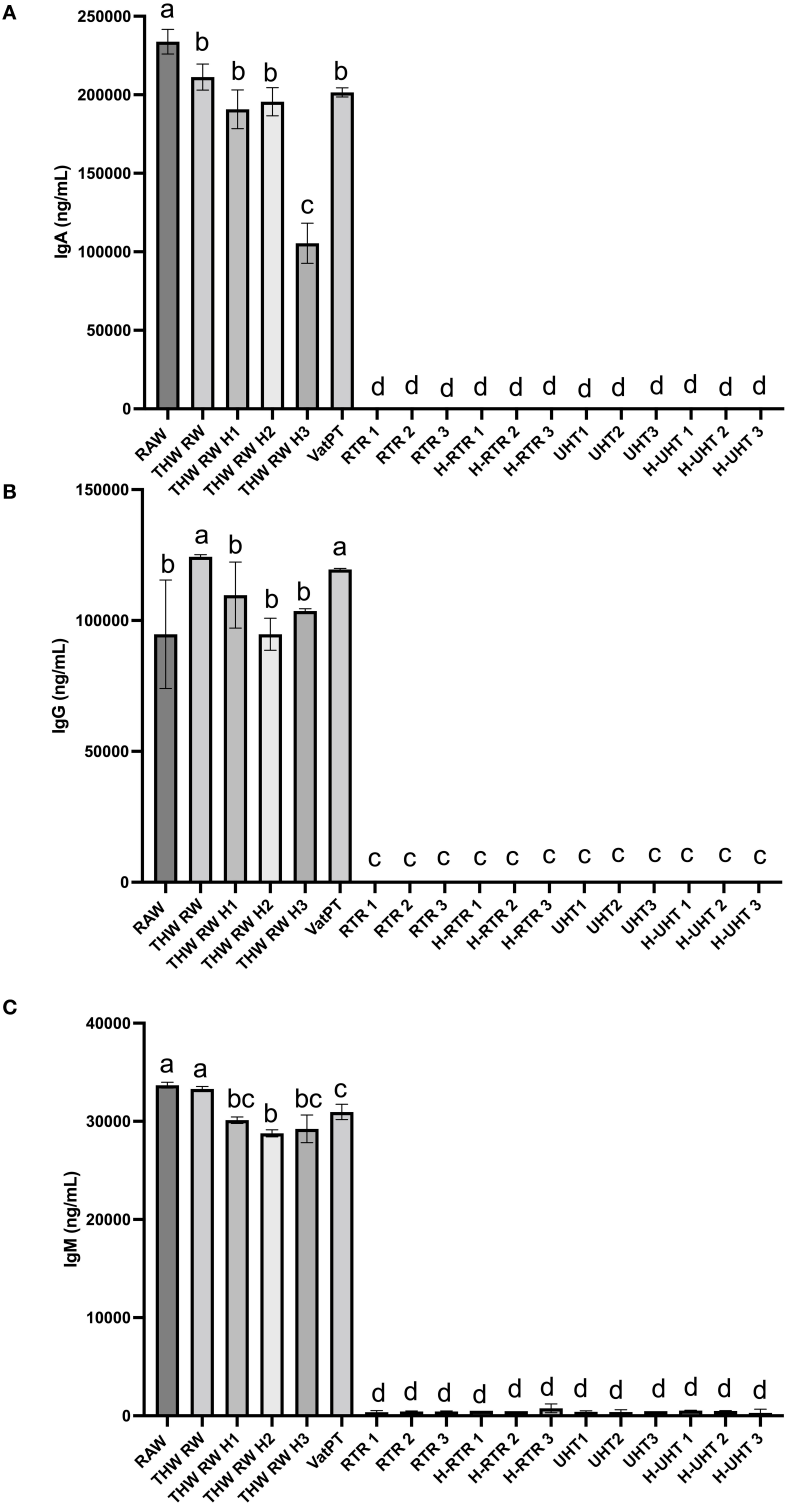
Concentrations of **(A)** IgA, **(B)** IgG, and **(C)** IgM in the donor human milk samples: raw milk (Raw), thawed raw milk (Thaw Raw), thawed and homogenized raw milk (Thaw Raw H1, Thaw Raw H2, and Thaw Raw H3), vat-pasteurized milk (Vat-PT), retort treated milk (RTR 1, 2, and 3), homogenized and retort treated milk (H-RTR 1, 2, and 3), ultra-high-temperature treated milk (UHT 1, 2, and 3), and homogenized and ultra-high-temperature treated milk (H-UHT 1, 2, and 3). One-way analysis of variance (ANOVA) with *post hoc* Tukey's tests were used to identify significant (*p* < 0.05) differences between treatments, denoted by letters.

The effect of different processing methods on the IgA, IgG, and IgM content of human milk was determined (Figure 2). Thawing, thawing plus homogenization and Vat-PT slightly decreased IgA and IgG content but not IgG content. All three immunoglobulins in the milk samples cannot be detected when RTR and UHT with or without homogenization were applied.

### Effect of processing methods on the concentration of lactoferrin, lysozyme, α-lactalbuminand elastase in donor human milk

**Figure 3:**
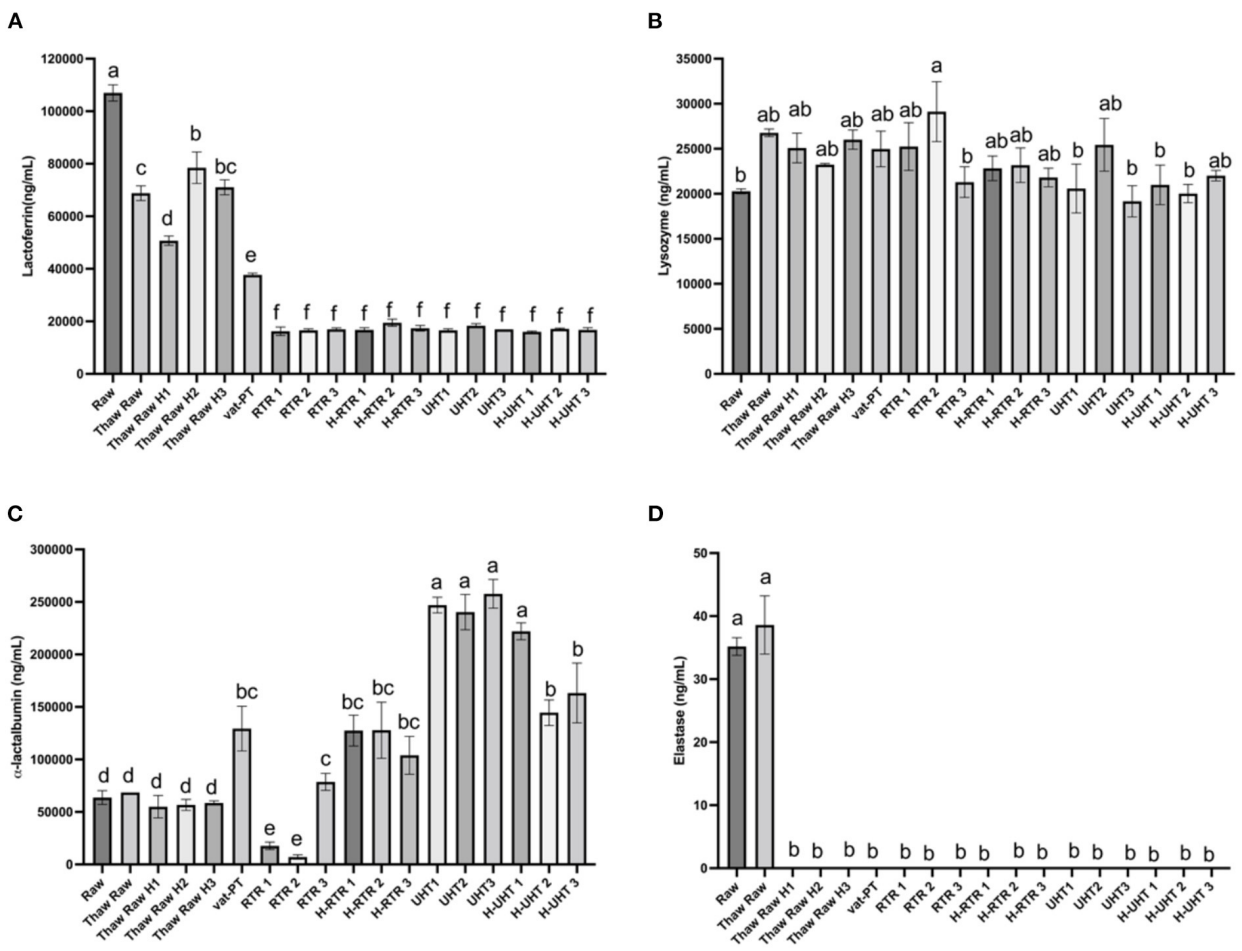
**(A)** Lactoferrin, **(B)** lysozyme, **(C)** α-lactalbumin, and **(D)** elastase concentrations in the donor human milk samples: raw milk (Raw), thawed raw milk (Thaw Raw), thawed and homogenized raw milk (Thaw Raw H1, Thaw Raw H2 and v H3), vat-pasteurized milk (Vat-PT), retort treated milk (RTR 1, 2, and 3), homogenized and retort treated milk (H-RTR 1, 2, and 3), ultra-high-temperature treated milk (UHT 1, 2, and 3), and homogenized and ultra-high-temperature treated milk (H-UHT 1, 2, and 3). One-way analysis of variance (ANOVA) with *post hoc* Tukey's tests were used to identify significant (*p* < 0.05) differences between treatments, denoted by letters.

The freeze-thaw process decreased donor milk lactoferrin immunoreactivity ~35% with little additional loss after thawing plus homogenization (on average <37% decrease in each of three batches) (Figure 3A). Around 65% of lactoferrin immunoreactivity was lost after Vat-PT. Both RTR and UHT processing reduced lactoferrin content 84% (Figure 3A). Homogenization following RTR or UHT treatment did not further decrease lactoferrin content (Figure 3A). Lysozyme content in human milk did not significantly change after any of the processing methods (Figure 3B).

α-Lactalbumin content did not change significantly after a freeze-thaw cycle with or without homogenization. However, Vat-PT, H-RTR, UHT and H-UHT increased α-lactalbumin concentration 160, 475, and 384%, respectively. RTR alone decreased α-lactalbumin 5% on average. This result is different from the visual density of bands in the SDS-PAGE, where the intensity of α-lactalbumin bands did not change much after Vat-PT and decreased after UHT, H-UHT, RTR and H-RTR.

Elastase content did not change after a freeze-thaw cycle but degraded to near zero ng/ml after thawing plus homogenization and after other heat treatments (Vat-PT, RTR and UHT).

### Effect of processing methods on the concentration of PIGR, osteopontin and TNFα in donor human milk

**Figure 4:**
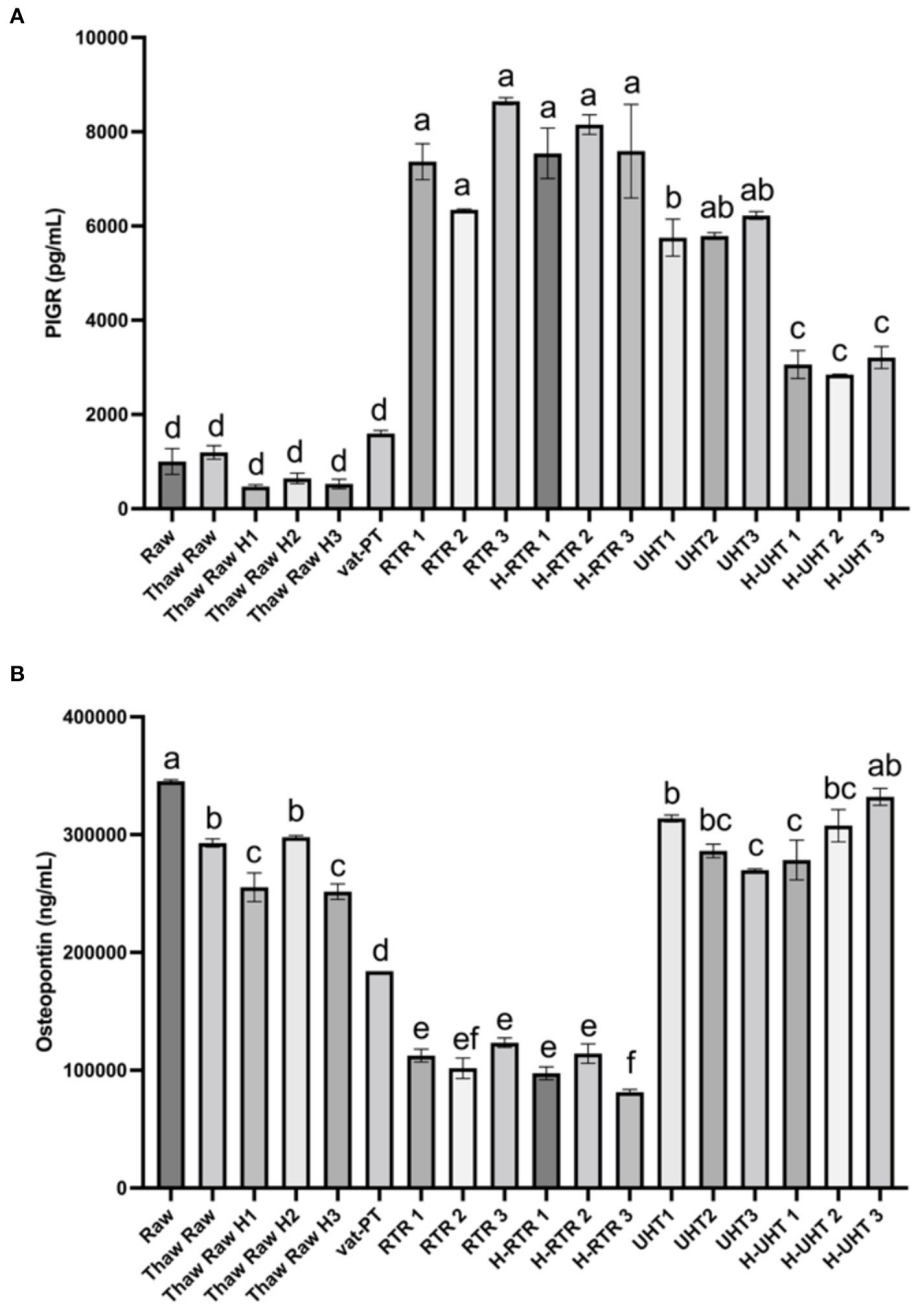
**(A)** PIGR and **(B)** osteopontin concentrations in the donor human milk samples: raw milk (Raw), thawed raw milk (Thaw Raw), thawed and homogenized raw milk (Thaw Raw H1, 2, and 3), vat-pasteurized milk (Vat-PT), retort treated milk (RTR 1, 2, and 3), homogenized and retort treated milk (H-RTR 1, 2, and 3), ultra-high-temperature treated milk (UHT 1, 2, and 3), and homogenized and ultra-high-temperature treated milk (H-UHT 1, 2, and 3). One-way analysis of variance (ANOVA) with *post hoc* Tukey's tests were used to identify significant (*p* < 0.05) differences between treatments, denoted by letters.

The effect of different processing methods on the PIGR content of donor milk was examined (Figure 4A). Thawing, thawing plus homogenization, and Vat-PT did not significantly alter PIGR concentration in human milk. PIGR content increased 7-fold after RTR and H-RTR and 4-fold after UHT processing. In contrast, UHT did not significantly alter osteopontin content, but retort processing decreased more than 70% of osteopontin in human milk (Figure 4B). Vat-PT also decreased osteopontin ~50% (Figure 4B). TNFα was not detected in any of the milk samples.

### Effect of processing methods on the concentration of VEGF, TGF-β1 and TGF-β2 in donor human milk

**Figure 5:**
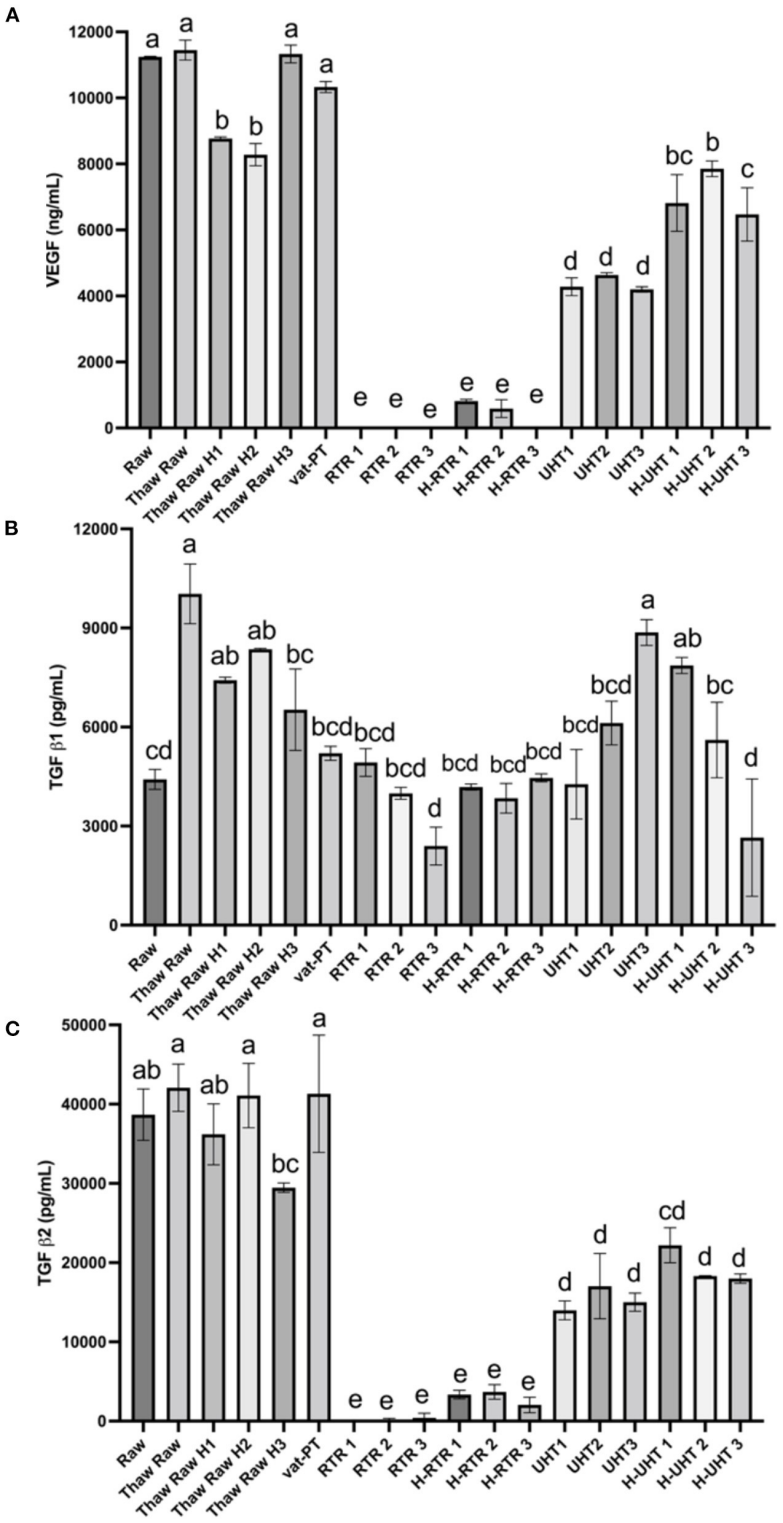
**(A)** VEGF, **(B)** TGF-β1, and **(C)** TGF-β2 concentrations in the donor human milk samples: raw milk (Raw), thawed raw milk (Thaw Raw), thawed and homogenized raw milk (Thaw Raw H1, 2, and 3), vat-pasteurized milk (Vat-PT), retort sterilization-treated milk (RTR 1, 2, and 3), homogenized and retort treated milk (H-RTR 1, 2, and 3), ultra-high-temperature treated milk (UHT 1, 2, and 3), and homogenized and ultra-high-temperature treated milk (H-UHT 1, 2, and 3). One-way analysis of variance (ANOVA) with *post hoc* Tukey's tests were used to identify significant (*p* < 0.05) differences between treatments, denoted by letters.

The effects of different processing methods on the content of these three growth factors in human milk were examined (Figure 5). Thawing, thawing plus homogenization and Vat-PT did not significantly change VEGF content. UHT and H-UHT decreased VEGF content 61 and 37%, respectively (Figure 5A). More than 95% of VEGF did not survive after retort or retort plus homogenization.

Similar trends were observed for processing effects on TGF-β2 as for effects on VEGF (Figure 5C). Thawing, thawing plus homogenization and Vat-PT did not change TGF-β2 significantly. UHT and H-UHT decreased TGF-β2 60 and 50%, respectively. H-RTR and RTR decreased TGF-β2 immunoreactivity 92 and 99%, respectively. TGF-β1 content was not changed significantly after thawing plus homogenization, Vat-PT, RTR, H-RTR, UHT or H-UHT. However, thawing alone significantly increased TGF-β1 content.

## Discussion

Donor milk banks and some commercial human milk processors use freezing and Vat-PT to ensure the microbiological safety of donor milk. Some commercial human milk processors use other techniques to process donor milk, including homogenization, RTR and UHT processing. Currently, donor milk processors lack information about the extent to which different processing techniques degrade or preserve bioactive milk proteins. Our study addresses this critical research gap as we examined the extent to which various treatments can preserve bioactive proteins. This information will support milk processors in determining how to optimally process donor milk to preserve specific milk proteins.

The result that the protein concentration of each infranatant of the 18 samples were lower than their corresponding whole sample likely resulted from centrifugation causing some milk proteins to move to the supernatant lipid layer or the precipitate. The result that the homogenized samples had the highest protein content indicated that homogenization increased protein content in the milk samples. One possible reason could be that homogenization broke down the large coagulated protein complexes into smaller units or caused unfolding of peptide chains and consequently allowed more surface contact between the peptides to reduce Cu^2+^ ions from the copper (II) sulfate in BCA reagent to Cu^1+^. Homogenization may also have decreased the interference of milk fat globules with the assay, either *via* interactions of reagents with the proteins or interference with light transmission. The finding that there was no significant difference between Raw, Vat-PT, Thaw Raw, Thaw Raw H, UHT and H-UHT in the infranatant (without lipid), but RTR and H-RTR had lower protein content compared with the other samples likely indicates that RTR processing led to increased protein coagulation, which increased protein losses in the precipitate after centrifugation.

SDS-PAGE was used to separate the proteins by molecular size and to show the relative abundances of major proteins in donor milk after processing by different methods. Five major groups of proteins, including immunoglobulins, lactoferrin, serum albumin, caseins and α-lactalbumin, were successfully separated and identified by SDS-PAGE. We found an extra band on the bottom of the lane in the samples with lipid compared with the samples without lipid. This band could represent milk fat globule-associated smaller proteins or smaller proteins removed during centrifugation. The SDS-PAGE data showed that the intensity of immunoglobulin bands did not decrease after thawing and Vat-PT, but that some bands (likely serum albumin and lactoferrin) that were in Raw milk samples disappeared after RTR, H-RTR, UHT, and H-UHT. The loss of these bands indicates protein degradation or aggregation. The loss of an additional band in RTR and H-RTR samples suggests that retort processing caused the greatest degradation of human milk proteins of the treatments studied herein. Our SDS-PAGE findings indicate, at least from a gel-based visual perspective, that milk proteins were preserved across Vat-PT, freezing and thawing, and homogenization, but degraded by retort and ultra-high-temperature processing.

Furthermore, the immunoreactivity of 13 bioactive proteins in donor milk were measured by ELISA. ELISA data also showed that the majority of IgA, IgG, and IgM immunoreactivity in these samples survived through the freeze-thaw cycle and Vat-PT but was completely lost after RTR and UHT. The IgA concentration in raw donor milk (~0.23 mg/ml) was similar to that reported by Goldblum et al. (26) and Chantry et al. (27)—both groups reported IgA concentration at ~0.37 mg/ml. Our study found that IgA concentration decreased ~10% after Vat-PT (62.5°C for 30 min), which is a little less than previous studies that reported a 21% loss of IgA (28). The results suggest that infants fed vat-pasteurized milk would still receive substantial passive immunoprotection. In contrast, infants fed RTR- and UHT-treated donor milk would lack the provision of intact immunoglobulins. Human milk immunoglobulins delivered to infants during breastfeeding are crucial in shaping and modulating immature infants' immune system and provide efficient protection against pathogens (29). Therefore, it may be important to provide infants who receive RTR- and UHT-processed donor milk with immunoglobulin supplementation to achieve similar health outcomes. A potential limitation is that measuring IgA concentration *via* ELISA does not necessarily reflect its activity or function.

The SDS-PAGE data show that the intensity of the lactoferrin band was lower after thawing and Vat-PT, even lower after UHT and H-UHT, and that the band was absent after RTR and H-RTR. The ELISA data also indicate 65% of the lactoferrin immunoreactivity was lost after Vat-PT, and more than 80% of lactoferrin activity was lost after UHT, H-UHT, RTR, and H-RTR. A previous study reported that 78% of lactoferrin immunoreactivity was lost after Vat-PT (24). Lactoferrin is the second most abundant protein in human milk. It prevents infection, plays a role in iron metabolism, has anti-inflammatory properties and is an antioxidant. Our data suggest that infants fed RTR- and UHT-processed donor milk receive significantly less intact, nondenatured lactoferrin compared with infants fed Vat-PT-processed donor milk.

α-Lactalbumin is the most abundant protein in human milk, comprising up to 25% of total protein (30). The SDS-PAGE data show that the intensity of the α-lactalbumin band was not changed with a freeze-thaw cycle, Vat-PT, UHT or H-UHT, but it was decreased by the RTR process. In contrast, the ELISA data indicate that Vat-PT, H-RTR, UHT, and H-UHT significantly increase α-lactalbumin immunoreactivity. RTR slightly decreases α-lactalbumin immunoreactivity. The ELISA data are consistent with data in a previous report by Elfagm and Wheelock (31) that the amount of immunoreactive α-lactalbumin detected in human milk increased after heating at 80°C for 5 min. Another study also reported that α-lactalbumin immunoreactivity increased after UHT (32). A similar result was observed for PIGR. The increased α-lactalbumin and PIGR activity after heat treatment could be due to heat-induced protein-protein interactions and configuration changes. In general, heating processes have potential to alter protein structure, including unfolding of secondary structure, cleavage and rearrangement of disulfide bonds, formation of new intermolecular interactions and the formation of aggregates (31, 33). Previous studies demonstrated that UHT processing caused the unfolding of antiparallel β-sheet and α-helix that are present in native α-lactalbumin (32). We hypothesize that the configuration change of α-lactalbumin during heating allows it to become more sensitive to binding to the antigens coated on the ELISA plate, thus showing an increased activity detected by ELISA.

Human milk contains numerous growth factors that likely impact the development of the infant intestinal tract, vasculature, nervous system and endocrine system. Herein, we measured three growth factors in donor milk–VEGF, TGF-β1 and TGF-β2. VEGF is a homodimeric 34–42-kD heparin-binding glycoprotein in human milk with potent angiogenic, mitogenic and vascular permeability-enhancing activities. TGF-β1 and TGF-β2 play a role in immune regulation in the developing infant. These three growth factors have similar heat sensitivity based on the results we observed. Vat-PT preserves all the immunoreactivity of these three growth factors. The Vat-PT result is consistent with the findings of a previous study showing that TGF-β2 survived with relatively little loss after heating at 56.9°C for 30 min (34). The present study is the first to compare the effects of the thawing, homogenization, Vat-PT, UHT and RTR on the activity of these three growth factors in donor milk.

This study provides immediately translatable information that can be used to inform milk handling processes in donor milk banks and by human milk processing companies. Milk processors can select processing techniques that best retain key bioactive proteins. Alternatively, bioactive proteins shown to be decreased by a specific treatment could be supplemented back into donor milk to maintain optimal bioactive protein provision to infants. Our findings provide information to supply infants with donor milk that better matches mother's milk in supporting their growth and development. This work provides essential preliminary data for future clinical studies examining how alternative donor milk processing techniques can impact infant health. Future research should determine the impact of other emerging processing techniques such as high-pressure processing and ultraviolet-C processing on the bioactive protein concentrations and activity in donor milk.

## Data availability statement

The original contributions presented in the study are included in the article/Supplementary material, further inquiries can be directed to the corresponding author.

## Author contributions

NL, JK, BK, DB, and DD designed research. NL, GO, and JK conducted research. NL analyzed data. NL, JK, and DD wrote the article. DD had primary responsibility for final content. All authors contributed to the article and approved the submitted version.

## Funding

This is study is supported by Prolacta Bioscience.

## Conflict of interest

The authors declare that this study received funding from Prolacta Bioscience. The funder had the following involvement with the study: collected the human milk samples, performed pooling of the samples, and conducted Vat-PT.

## Publisher's note

All claims expressed in this article are solely those of the authors and do not necessarily represent those of their affiliated organizations, or those of the publisher, the editors and the reviewers. Any product that may be evaluated in this article, or claim that may be made by its manufacturer, is not guaranteed or endorsed by the publisher.
