# Corrigendum: Structural and functional changes of bioactive proteins in donor human milk treated by vat-pasteurization, retort sterilization, ultra-high-temperature sterilization, freeze-thawing and homogenization

**DOI:** 10.3389/fnut.2024.1371799

**Published:** 2024-03-20

**Authors:** Ningjian Liang, Jeewon Koh, Bum Jin Kim, Gulustan Ozturk, Daniela Barile, David C. Dallas

**Affiliations:** ^1^Nutrition Program, School of Biological and Population Health Sciences, College of Public Health and Human Sciences, Oregon State University, Corvallis, OR, United States; ^2^Department of Food Science and Technology, University of California, Davis, Davis, CA, United States

**Keywords:** heat treatment, pressure, microbiological safety, lactation, preterm infant

In the published article, there was a grammatical error.

A correction has been made to **Introduction**, paragraph three. This sentence previously stated:

“Consuming donor milk provided by milk banks and by donor milk processing companies is an alternative option for preterm infants whose mothers are absence postpartum”

The corrected sentence appears below:

“Consuming donor milk provided by milk banks and by donor milk processing companies is an alternative option for preterm infants whose parents cannot produce sufficient parent's own milk.”

The authors apologize for this error and state that this does not change the scientific conclusions of the article in any way. The original article has been updated.

