# Supplementary material for: Structural and functional changes of bioactive proteins in donor human milk treated by vat-pasteurization, retort sterilization, ultra-high-temperature sterilization, freeze-thawing and homogenization
Source: Front Nutr. 2022 Sep 15;9:926814. doi: 10.3389/fnut.2022.926814 (PMC9521613; doi:10.3389/fnut.2022.926814)

Supplementary Material

**Supplemental TABLE 1.** Protein concentration in whole pooled donor human milk samples and infranatants after various processing^1^

|  | **Protein concentration (mg/mL)** | | | | | |
| --- | --- | --- | --- | --- | --- | --- |
|  | Whole sample (with lipid) | | | Infranatant (without lipid) | | |
| Raw | 10.7^cd^ ± 0.1 | | | 10.0^a^ ± 0.1 | | |
| Vat-PT | 9.7^d^ ± 0.1 | | | 9.5^ab^ ± 0.2 | | |
| Thaw Raw | 11.3^bcd^ ± 0.0 | | | 8.2^abc^ ± 0.2 | | |
| Thaw Raw H | 1 | 13.5 ± 0.2 | 14.0^ab^ ± 0.5 | 1 | 8.9 ± 0.2 | 9.1^abc^ ± 0.3 |
|  | 2 | 14.1 ± 0.3 |  | 2 | 9.2 ± 0.1 |  |
|  | 3 | 14.5 ± 0.3 |  | 3 | 9.1 ± 0.4 |  |
| UHT | 1 | 12.4 ± 0.2 | 11.9^abcd^ ± 1.5 | 1 | 10.3 ± 0.3 | 9.7^a^ ± 1.3 |
|  | 2 | 13.3 ± 0.1 |  | 2 | 10.7 ± 0.3 |  |
|  | 3 | 9.9 ± 0.1 |  | 3 | 8.1 ± 0.3 |  |
| H-UHT | 1 | 12.6 ± 0.1 | 13.0^abc^ ± 0.9 | 1 | 7.6 ± 0.2 | 7.9^abc^ ± 0.5 |
|  | 2 | 12.1 ± 0.1 |  | 2 | 7.8 ± 0.3 |  |
|  | 3 | 14.2 ± 0.2 |  | 3 | 8.5 ± 0.2 |  |
| RTR | 1 | 6.7 ± 0.2 | 9.8^d^ ± 2.9 | 1 | 5.4 ± 0.3 | 7.0^bc^ ± 2.4 |
|  | 2 | 9.5 ± 0.3 |  | 2 | 5.3 ± 0.2 |  |
|  | 3 | 13.4 ± 0.3 |  | 3 | 10.2 ± 0.4 |  |
| H-RTR | 1 | 15.0 ± 0.6 | 14.4^a^ ± 1.0 | 1 | 8.5 ± 0.4 | 6.5^c^ ± 1.5 |
|  | 2 | 13.2 ± 0.2 |  | 2 | 5.8 ± 0.0 |  |
|  | 3 | 15.1 ± 0.1 |  | 3 | 5.2 ± 0.1 |  |

^1^The data are expressed as average ± SD. One-way analysis of variance (ANOVA) with post hoc Tukey’s tests were used to identify significant (*p*<0.05) differences between treatments, denoted by letters. Uncommon letters represent that they are significantly different and common letters represent that they are not significantly different from each other. Raw milk (Raw), thawed raw milk (Thaw Raw), thawed and homogenized raw milk (Thaw Raw H 1, Thaw Raw H 2 and Thaw Raw H 3), vat-pasteurized milk (Vat-PT), retort treated milk (RTR 1, 2 and 3), homogenized and retort treated milk (H-RTR 1, 2 and 3), ultra-high-temperature treated milk (UHT 1, 2 and 3), homogenized and ultra-high-temperature treated milk (H-UHT 1, 2 and 3).

**Supplemental Figure 1.** The diagram illustrates the processing conditions and labelling of the processed milk samples.

**
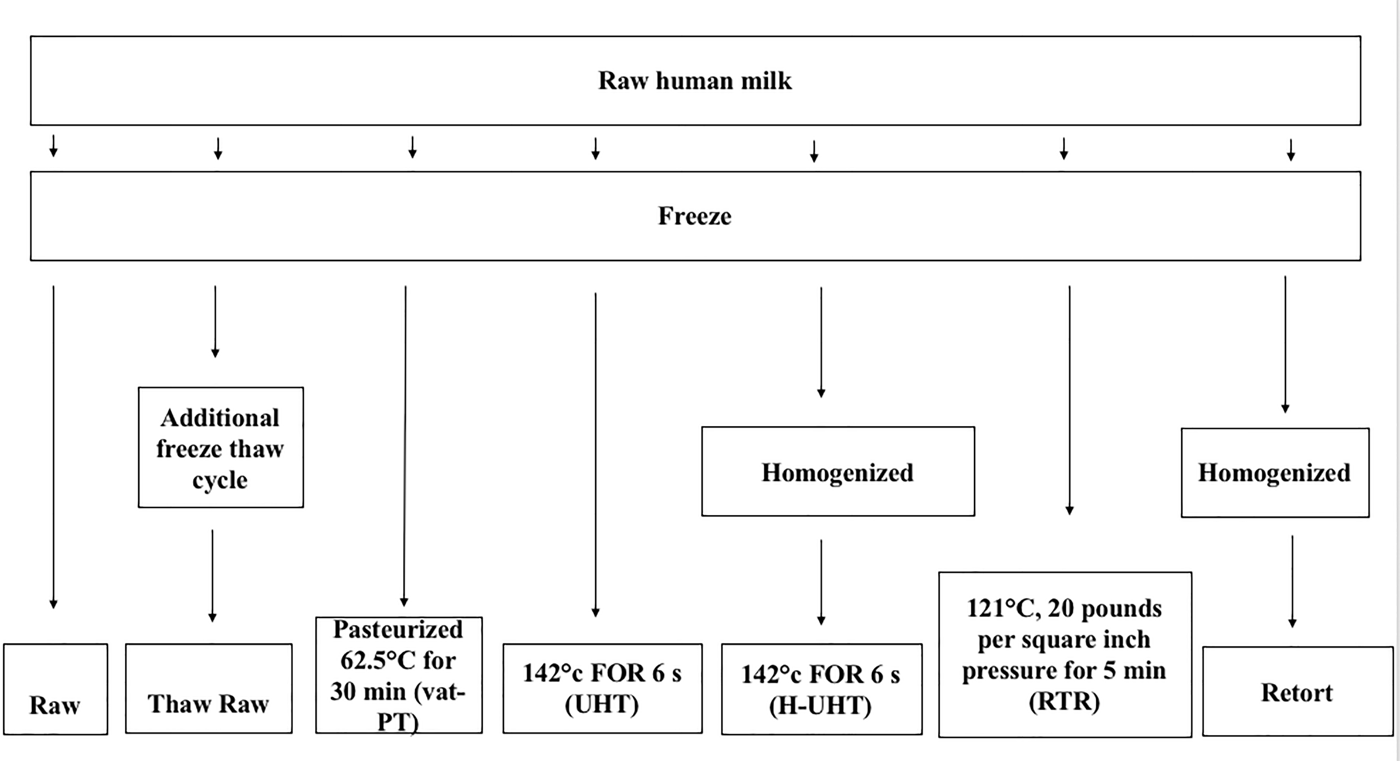
**

**Supplemental Figure 2.** SDS-PAGE of raw milk (Raw), retort treated milk (RTR 1, RTR 2, RTR 3), homogenized and retort treated milk (H-RTR 1, H-RTR 2, H-RTR 3) with lipid (w/L) and without lipid (w/o L). The unit of the y-axis is kDa.


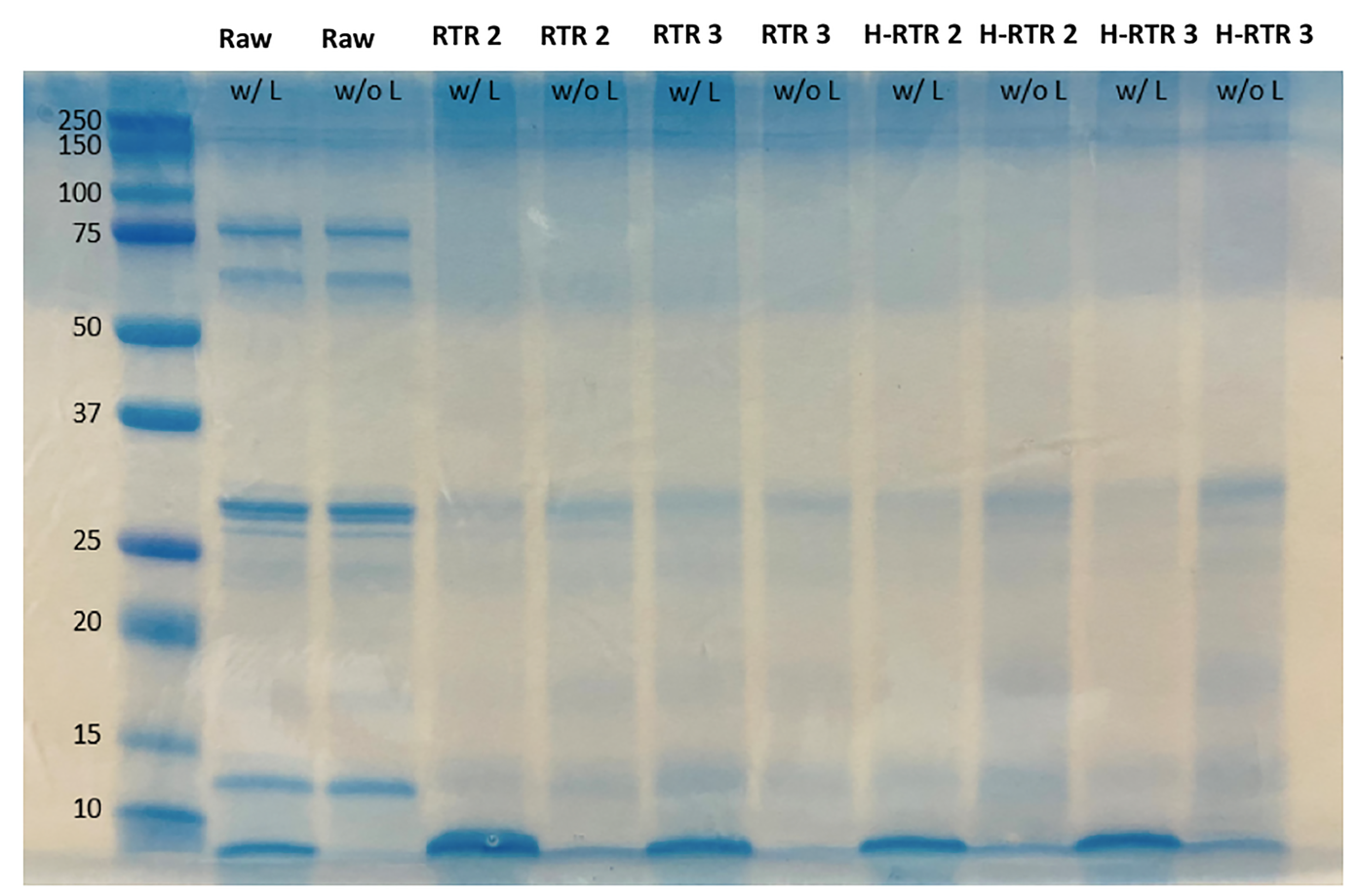


**Supplemental Figure 3**. SDS-PAGE of thawed and homogenized raw milk (Thaw Raw H1, Thaw Raw H2 and Thaw Raw H3), ultra-high-temperature processed milk (UHT 1, UHT 2 and UHT 3), homogenized and ultra-high-temperature processed milk (H-UHT 1, H-UHT 2, H-UHT 3), retort processed milk (RTR 1), homogenized and retort processed milk (H-RTR 1). The unit of the y-axis is kDa.


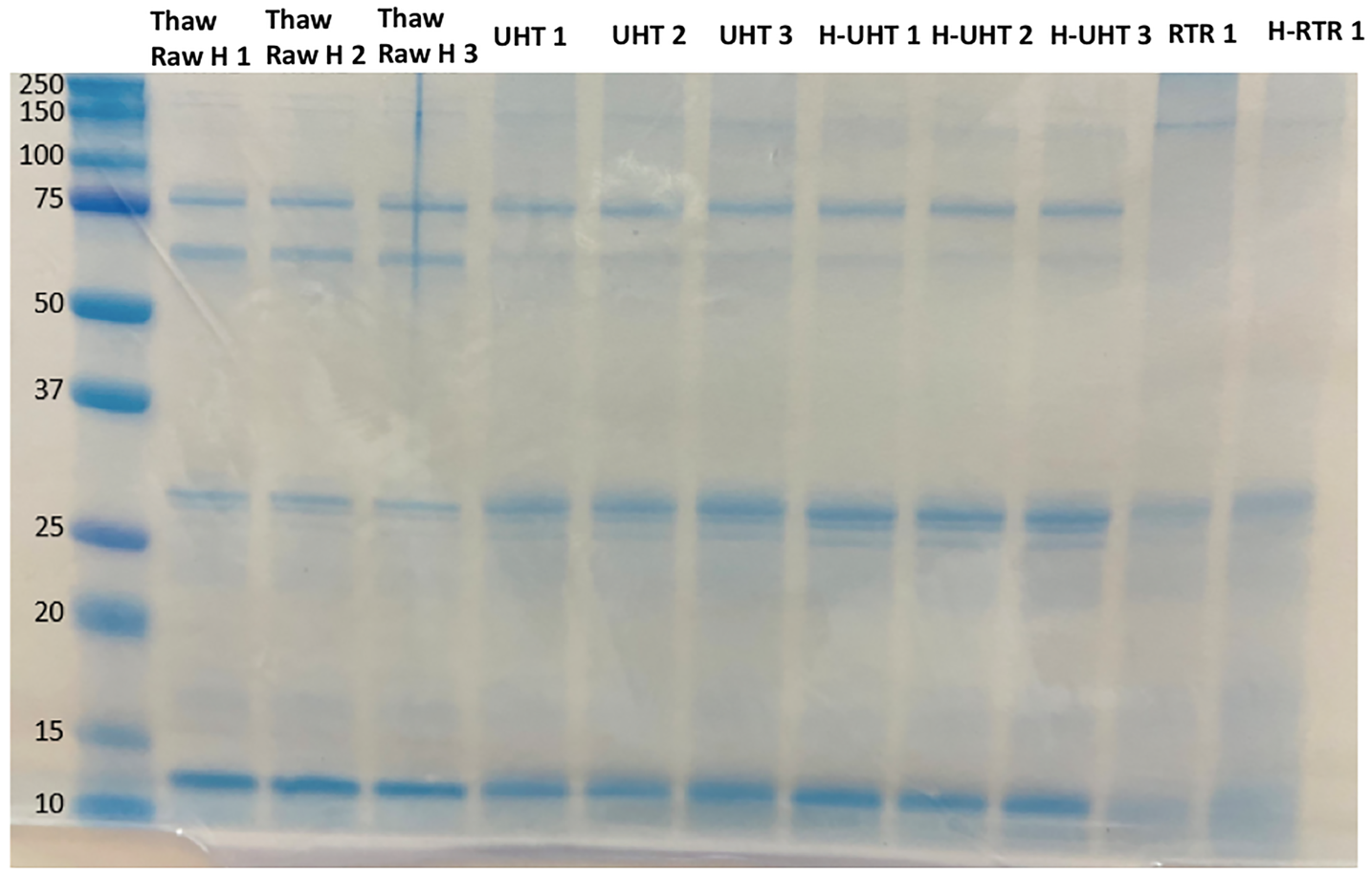

Supplement: Supplementary file 1 [file Data_Sheet_1.docx]
